# Supplementary material for: Sociodemographic and geographic determinants of childhood immunization coverage and equity in Ghana: Analysis of the 2022 demographic and health survey
Source: PLoS One. 2026 Apr 15;21(4):e0337505. doi: 10.1371/journal.pone.0337505 (PMC13082596; doi:10.1371/journal.pone.0337505)
Supplement: Attachment — Submitted filename: kw comments.pdf [file pone.0337505.s001.pdf]

PLOS ONE

Sociodemographic and Geographic Determinants of Childhood Immunization Coverage and Equity in Ghana: Analysis of the 2022 Demographic and Health Survey

--Manuscript Draft--

**Manuscript Number:**	PONE-D-25-59990
**Article Type:**	Research Article
**Full Title:**	Sociodemographic and Geographic Determinants of Childhood Immunization Coverage and Equity in Ghana: Analysis of the 2022 Demographic and Health Survey
**Short Title:**	Determinants of Childhood Immunization Coverage and Equity in Ghana
**Corresponding Author:**	Ahlam Tunteeya Saani, MD<br>University of Ghana Dental School: University of Ghana Medical School<br>Accra, GHANA
**Keywords:**	Childhood immunization, vaccination coverage, health equity, immunization dropout, Ghana, Demographic and Health Survey, sub-Saharan Africa, vaccine timeliness
**Abstract:**	Background<br><br>Childhood immunization remains a cornerstone of public health in sub-Saharan Africa, yet substantial gaps in coverage, equity, and schedule adherence persist. This study examines immunization coverage, sociodemographic disparities, predictors of completion, and policy implementation effectiveness in Ghana using nationally representative data.<br><br>Methods<br><br>Data from 3,787 children aged 12 to 35 months in the 2022 Ghana Demographic and Health Survey were analysed using stratified two-stage cluster sampling across 613 enumeration areas. Weighted coverage estimates were calculated for individual vaccines and composite indicators including full immunization, dropout rates, and timeliness. Design-adjusted chi-square tests identified bivariate associations between sociodemographic factors and immunization outcomes. Multivariate logistic regression models incorporated sociodemographic characteristics, healthcare utilization, and information access to identify independent predictors. Equity analysis employed concentration indices, rate ratios, and intersectional stratification.<br><br>Findings<br><br>Full immunization coverage reached 63.9% (95% CI: 61.7 to 66.1%), with 29.3% partial immunization and 4.9% zero-dose children. Substantial inequalities emerged across wealth (17.4 percentage point gap), maternal education (17.2 percentage point gap), and region (32.0 percentage point gap). The concentration index of 0.061 indicated modest pro-rich inequality. Dropout rates were concerning, particularly 37.5% attrition between measles-rubella first and second doses. In adjusted models, facility delivery (aOR=1.81, 95% CI: 1.37 to 2.40), four or more antenatal care visits (aOR=1.64, 95% CI: 1.26 to 2.13), higher wealth (aOR=1.43, 95% CI: 1.07 to 1.91), and maternal secondary or higher education (aOR=1.35, 95% CI: 1.05 to 1.73) independently predicted full immunization.<br><br>Interpretation<br><br>Ghana's immunization program achieves broad initial access but faces critical challenges in retention, timeliness, and equity. Policy priorities should emphasize completion-focused interventions including tracking systems, reminder mechanisms, and targeted outreach to disadvantaged populations.
**Order of Authors:**	Ahlam Tunteeya Saani, MD
	Goldfield Edem Azumah, MPH
**Additional Information:**	

Powered by Editorial Manager® and ProduXion Manager® from Aries Systems Corporation

Question	Response
**Financial Disclosure**<br><br>Enter a financial disclosure statement that describes the sources of funding for the work included in this submission. Review the submission guidelines for detailed requirements. View published research articles from *PLOS ONE* for specific examples.<br><br>This statement is required for submission and **will appear in the published article** if the submission is accepted. Please make sure it is accurate.<br><br>**Funded studies**<br>Enter a statement with the following details:<br>• Initials of the authors who received each award<br>• Grant numbers awarded to each author<br>• The full name of each funder<br>• URL of each funder website<br>• Did the sponsors or funders play any role in the study design, data collection and analysis, decision to publish, or preparation of the manuscript?<br><br>Did you receive funding for this work?	The author(s) received no specific funding for this work.
**Competing Interests**<br><br>Use the instructions below to enter a competing interest statement for this submission. On behalf of all authors, disclose any competing interests that could be perceived to bias this work—acknowledging all financial support and any other relevant financial or non-financial competing interests.<br><br>This statement is required for submission and **will appear in the published article** if the submission is accepted. Please make sure it is accurate and that any funding sources listed in your Funding Information later in the submission form are also	The authors have declared that no competing interests exist.

declared in your Financial Disclosure statement.<br><br>View published research articles from *PLOS ONE* for specific examples.<br><br>**NO authors have competing interests**<br><br>Enter: *The authors have declared that no competing interests exist.*<br><br>**Authors with competing interests**<br><br>Enter competing interest details beginning with this statement:<br><br>*I have read the journal's policy and the authors of this manuscript have the following competing interests: [insert competing interests here]*<br><br>* typeset	
**Ethics Statement**<br><br>Enter an ethics statement for this submission. This statement is required if the study involved:<br><br>• Human participants<br>• Human specimens or tissue<br>• Vertebrate animals or cephalopods<br>• Vertebrate embryos or tissues<br>• Field research<br><br>Write "N/A" if the submission does not require an ethics statement.<br><br>General guidance is provided below. Consult the submission guidelines for detailed instructions. **Make sure that all information entered here is included in the Methods section of the manuscript.**	This study involved secondary analysis of de-identified, publicly available data from the Ghana 2022 Demographic and Health Survey. Ethical approval for the original data collection was obtained by the Ghana Health Service Ethical Review Committee and the Institutional Review Board of ICF International. Written informed consent was obtained from all survey participants during the original data collection. As this study involved analysis of existing de-identified datasets available in the public domain, additional institutional ethics approval was not required. Access to the dataset was granted by the DHS Program following standard registration procedures at dhsprogram.com.

**Format for specific study types**<br><br>**Human Subject Research (involving human participants and/or tissue)**<br>• Give the name of the institutional review board or ethics committee that approved the study<br>• Include the approval number and/or a statement indicating approval of this research<br>• Indicate the form of consent obtained (written/oral) or the reason that consent was not obtained (e.g. the data were analyzed anonymously)<br><br>**Animal Research (involving vertebrate animals, embryos or tissues)**<br>• Provide the name of the Institutional Animal Care and Use Committee (IACUC) or other relevant ethics board that reviewed the study protocol, and indicate whether they approved this research or granted a formal waiver of ethical approval<br>• Include an approval number if one was obtained<br>• If the study involved *non-human primates*, add *additional details* about animal welfare and steps taken to ameliorate suffering<br>• If anesthesia, euthanasia, or any kind of animal sacrifice is part of the study, include briefly which substances and/or methods were applied<br><br>**Field Research**<br><br>Include the following details if this study involves the collection of plant, animal, or other materials from a natural setting:<br>• Field permit number<br>• Name of the institution or relevant body that granted permission	
**Data Availability**<br><br>Authors are required to make all data underlying the findings described fully available, without restriction, and from the time of publication. PLOS allows rare exceptions to address legal and ethical concerns. See the PLOS Data Policy and FAQ for detailed information.	Yes - all data are fully available without restriction

Powered by Editorial Manager® and ProduXion Manager® from Aries Systems Corporation

A Data Availability Statement describing where the data can be found is required at submission. Your answers to this question constitute the Data Availability Statement and **will be published in the article**, if accepted.<br><br>**Important:** Stating 'data available on request from the author' is not sufficient. If your data are only available upon request, select 'No' for the first question and explain your exceptional situation in the text box.<br><br>Do the authors confirm that all data underlying the findings described in their manuscript are fully available without restriction?	
**Describe where the data may be found in full sentences. If you are copying our sample text, replace any instances of XXX with the appropriate details.**<br><br>• If the data are **held or will be held in a public repository**, include URLs, accession numbers or DOIs. If this information will only be available after acceptance, indicate this by ticking the box below. For example: *All XXX files are available from the XXX database (accession number(s) XXX, XXX.).*<br>• If the data are all contained **within the manuscript and/or Supporting Information files**, enter the following: *All relevant data are within the manuscript and its Supporting Information files.*<br>• If neither of these applies but you are able to provide **details of access elsewhere**, with or without limitations, please do so. For example:<br><br>*Data cannot be shared publicly because of [XXX]. Data are available from the XXX Institutional Data Access / Ethics Committee (contact via XXX) for researchers who meet the criteria for access to confidential data.*<br><br>*The data underlying the results presented in the study are available from (include the name of the third party*	The data underlying this study are publicly available from the Demographic and Health Surveys (DHS) Program. The Ghana 2022 Demographic and Health Survey dataset can be accessed through the DHS Program website at dhsprogram.com following completion of a brief registration process. Interested researchers must register for a free account on the DHS Program website and submit a data request describing their research objectives. Upon approval of the data request by the DHS Program, which typically occurs within 24-48 hours for legitimate research purposes, the dataset can be downloaded at no cost. The authors did not receive any special access privileges to the data, and other researchers can access the same dataset through the standard DHS Program data request procedures. The specific dataset used in this study is the Ghana Demographic and Health Survey 2022 (dataset file: GHIR8BDT.DTA for children's records and GHIR8BFL.DTA for women's records). All data access and use must comply with the DHS Program's data use terms and conditions, which require proper citation of the data source and restrict redistribution of the raw datasets to third parties.

and contact information or URL).<br>• This text is appropriate if the data are owned by a third party and authors do not have permission to share the data.<br><br>* typeset	
Additional data availability information:	

Powered by Editorial Manager® and ProduXion Manager® from Aries Systems Corporation

Manuscript
Click here to access/download;Manuscript;Ghana Childhood Immunization Coverage and Equity in.docx

Sociodemographic and Geographic Determinants of Childhood Immunization Coverage and Equity in Ghana: Analysis of the 2022 Demographic and Health Survey

Short Title: Determinants of Childhood Immunization Coverage and Equity in Ghana

Ahlam Tuneteeya Saani, BDS, MPH[1] and Goldfield Edem Azumah, MPH[2]

[1]University of Ghana Dental School, College of Health Sciences, University of Ghana

[2]Korle Bu Teaching Hospital, Accra

Corresponding author: Ahlam Tuneteeya Saani, University of Ghana Dental School, College of Health Sciences, University of Ghana. Email: ahlamsaani@icloud.com

Abstract

Background: Childhood immunization remains a cornerstone of public health in sub-Saharan Africa, yet substantial gaps in coverage, equity, and schedule adherence persist. This study examines immunization coverage, sociodemographic disparities, predictors of completion, and policy implementation effectiveness in Ghana using nationally representative data.

Methods: Data from 3,787 children aged 12 to [illegible] months in the 2022 Ghana Demographic and Health Survey were analysed using stratified two-stage cluster sampling across 613 enumeration areas. Weighted coverage estimates were calculated for individual vaccines and composite indicators including full immunization, dropout rates, and timeliness. Design-adjusted chi-square tests identified bivariate associations between sociodemographic factors and immunization outcomes. Multivariate logistic regression models incorporated sociodemographic characteristics, healthcare utilization, and information access to identify independent predictors. Equity analysis employed concentration indices, rate ratios, and intersectional stratification.

Findings: Full immunization coverage reached 63.9% (95% CI: 61.7 to 66.1%), with 29.3% partial immunization and 4.9% zero-dose children. Substantial inequalities emerged across wealth (17.4 percentage point gap), maternal education (17.2 percentage point gap), and region (32.0 percentage point gap). The concentration index of 0.061 indicated modest pro-rich inequality. Dropout rates were concerning, particularly 37.5% attrition between measles-rubella first and second doses. In adjusted models, facility delivery (aOR=1.81, 95% CI: 1.37 to 2.40), four or more antenatal care visits (aOR=1.64, 95% CI: 1.26 to 2.13), higher wealth (aOR=1.43, 95% CI: 1.07 to 1.91), and maternal secondary or higher education (aOR=1.35, 95% CI: 1.05 to 1.73) independently predicted full immunization.

Interpretation: Ghana's immunization program achieves broad initial access but faces critical challenges in retention, timeliness, and equity. Policy priorities should emphasize completion-focused interventions including tracking systems, reminder mechanisms, and targeted outreach to disadvantaged populations.

Funding: None.

Introduction

Vaccination stands as one of public health's most effective interventions, having prevented millions of deaths and nearly eradicated diseases. [1] However, substantial gaps persist in achieving universal coverage, ensuring schedule completion, reaching disadvantaged populations equitably, and administering vaccines at age-appropriate intervals that optimize protection during vulnerable periods. [2,3]

Sub-Saharan Africa faces challenges in immunization delivery despite sustained investments and policy commitments. [4] The region accounts for a disproportionate share of unimmunized and under-immunized children globally, with coverage plateaus and recent reversals threatening progress toward Sustainable Development Goal targets and the Immunization Agenda 2030 objectives. [4] Persistent inequalities in coverage across socioeconomic, geographic, and demographic dimensions indicate that universal policy frameworks have not translated into equitable service delivery. [5] Dropout rates between vaccine doses remain elevated, timeliness of administration frequently deviates from recommended schedules, and health system weaknesses including stock-outs, inadequate cold chain capacity, and insufficient healthcare worker training compromise programme effectiveness. [6]

Ghana has made substantial progress in childhood immunization over the past two decades, with coverage for traditional vaccines increasing from below 60% in the early 2000s to above 80% for most antigens by the 2020s. [7] The country has successfully introduced multiple newer vaccines including pneumococcal conjugate vaccine in 2012, rotavirus vaccine in 2012, and measles-rubella second dose in 2018, demonstrating programmatic capacity for innovation. [7] Ghana's Expanded Programme on Immunization operates through an integrated health service delivery platform

combining facility-based services with outreach activities, supported by the Ghana Health Service district health management structures. [7]

Despite Ghana's progress, substantial knowledge gaps remain regarding the patterns, determinants, and equity dimensions of immunization coverage using population-based data that can validate administrative statistics. Previous analyses have often focused on single dimensions such as urban-rural disparities or wealth-related inequalities in isolation, without examining how multiple disadvantages intersect to create particularly vulnerable populations. [7, 9-12] Limited attention has been given to retention through multi-dose series and across the complete schedule, with most analyses emphasizing initiation rather than completion. Timeliness of vaccination has received inadequate attention in Ghana and broader sub-Saharan African contexts due to data quality challenges with vaccination dates.

This study addresses these knowledge gaps through comprehensive analysis of immunization coverage, disparities, determinants, and policy implementation using data from the 2022 Ghana Demographic and Health Survey. The research objectives encompass documenting coverage patterns for individual vaccines and composite indicators including full immunization, partial immunization, and zero-dose prevalence, while examining timeliness of vaccine administration relative to recommended schedules. The analysis identifies disparities across sociodemographic characteristics including residence, wealth, maternal education, region, and intersectional categories, and determines independent predictors of immunization completion through multivariate regression. The study assesses equity using multiple complementary inequality measures, analyzes dropout patterns identifying critical retention challenges, and evaluates policy implementation effectiveness against national targets. By examining these dimensions using rigorous analytical methods applied to nationally representative data, this study aims to provide

evidence-based guidance for strengthening Ghana's immunization programme and inform similar efforts in comparable sub-Saharan African settings.

Materials and Methods

Study Design and Data Source

This study analysed data from the 2022 Ghana Demographic and Health Survey, a nationally representative cross-sectional household survey conducted between October 17, 2022, and January 14, 2023, by the Ghana Statistical Service in collaboration with the Ghana Health Service and ICF International. The survey employed a stratified two-stage cluster sampling design. In the first stage, 613 enumeration areas serving as clusters were selected with probability proportional to size from the 2021 Ghana Population and Housing Census frame. In the second stage, systematic random sampling selected approximately 30 households per cluster, yielding representative samples across Ghana's 16 regions and urban-rural strata.

Study Population and Sample

The study population comprised children aged 12 to 35 months at the time of survey, representing those who had sufficient time to complete the full immunization schedule including the 18-month measles-rubella second dose while minimizing recall bias for older children. The analytic sample included 3,787 children with complete data on key variables. Children were eligible if they were usual household members or visitors present the night before the survey and had surviving mothers available for interview. This age range was selected to align with standard Demographic and Health Survey immunization coverage assessment protocols and enable international comparability.

Data Collection Procedures

Trained interviewers conducted face-to-face interviews with mothers or primary caregivers using structured questionnaires translated into major Ghanaian languages. For each child, interviewers requested to see the child's vaccination card. When cards were available, interviewers transcribed vaccination dates for all vaccines directly from the card. For children without cards or when cards were not available at the interview, mothers were asked to recall whether their child had received each vaccine. Detailed information on household characteristics, maternal demographics, healthcare utilization, media exposure, and other relevant factors was collected simultaneously. Survey protocols included standard data quality assurance measures throughout data collection.

Variable Definitions

The primary outcome was full immunization coverage, defined as receipt of one dose of Bacille Calmette-Guérin vaccine, three doses of pentavalent vaccine protecting against diphtheria, tetanus, pertussis, hepatitis B, and Haemophilus influenzae type b, three doses of pneumococcal conjugate vaccine, three doses of oral polio vaccine, two doses of rotavirus vaccine, first dose of measles-rubella vacci[illegible] and one dose of yellow fever vaccine. This definition aligns with Ghana's national immunization schedule and World Health Organization recommendations. Secondary outcomes included basic immunization coverage comprising traditional vaccines only, partial immunization defined as receipt of some but not all recommended vaccines, zero-dose status indicating receipt of no vaccines, individual vaccine coverage rates, dropout rates between doses, and timeliness of vaccination representing administration within recommended age windows.

Dropout rates were calculated as the proportion of children receiving an earlier vaccine who failed to receive a later vaccine in the same series using standard formulas. Timeliness was assessed among children with complete date information for both birth and vaccination, using Century Month Code conversion to calculate age at vaccination precisely. Timely vaccination windows

were defined as BCG within one month of birth, pentavalent first dose between 1.0 to 2.5 months, pentavalent third dose between 3.0 to 4.5 months, and measles-rubella first dose between 8 to 11 months of age.

Sociodemographic factors included residence categorized as urban versus rural, wealth status, maternal education, maternal age, child sex, and birth order. Wealth status was derived from the Demographic and Health Survey wealth index, calculated through principal components analysis of household assets, housing characteristics, and access to utilities and services. For multivariate analysis, wealth quintiles were collapsed into tertiles to improve statistical power while maintaining discrimination. Maternal education was categorized as no education, primary, secondary, and higher education levels.

Healthcare utilization variables captured maternal engagement with formal health services during pregnancy and childbirth. Antenatal care was dichotomized as four or more visits versus fewer visits. Place of delivery distinguished facility-based births from home births. Media exposure indicated whether mothers regularly accessed newspapers, radio, or television at least weekly.

Statistical Analysis

All analyses incorporated survey design features using R version 4.3.1 with the survey package for complex survey analysis. Sampling weights were applied to account for unequal selection probabilities and differential response rates. Variance estimates were adjusted for clustering within enumeration areas using Taylor series linearization. Weighted prevalence estimates with standard errors and 95% confidence intervals were calculated for all coverage indicators and sociodemographic characteristics.

Bivariate associations between potential predictors and full immunization status were assessed using design-adjusted Pearson chi-square tests. Multivariate logistic regression models were constructed using a progressive modeling strategy building from sociodemographic factors to healthcare utilization variables to information access and distance barriers. Adjusted odds ratios with 95% confidence intervals were calculated. Stratified analyses examined whether predictor associations differed across urban and rural settings.

Equity analysis employed concentration indices using the Kakwani formula based on wealth quintile ranking. Rate ratios comparing coverage between the richest and poorest quintiles provided relative inequality measures. Intersectional analysis examined coverage across combinations of residence, wealth, and education to identify the most advantaged and disadvantaged groups.

Ethical Considerations

The 2022 Ghana Demographic and Health Survey received ethical approval from the Ghana Health Service Ethical Review Committee and the ICF Institutional Review Board. All participants provided written informed consent before enrolment. This secondary analysis of de-identified publicly available data was determined to be exempt from additional ethical review.

Results

Sociodemographic Characteristics

The weighted analysis of 3,787 children aged 12 to 35 months revealed balanced distribution across urban and rural areas, with 48.6% residing in urban settings and 51.4% in rural areas. Representation across wealth quintiles was relatively even, with the poorest quintile comprising 23.1% and the richest 17.1% of the sample. Maternal characteristics demonstrated considerable

diversity, with mean maternal age of 29.7 years. Educational attainment showed 22.7% of mothers had no formal education, 21.6% completed primary education, 47.2% achieved secondary education, and 12.8% attained higher education.

Table 1: Sociodemographic Characteristics of Study Population

Weighted distribution of maternal, child, and household characteristics among children aged 12-35 months participating in the Ghana Demographic and Health Survey 2022

Characteristic	n (weighted)	Percentage / Mean	SE / SD
**SAMPLE CHARACTERISTICS**			
Total sample size	3,787	100.0	—
Number of clusters	613	—	—
Number of strata	32	—	—
**MATERNAL CHARACTERISTICS**			
Maternal age (years), mean ± SD	—	29.7	±6.3
Maternal age groups (years)			

**Characteristic**	**n (weighted)**	**Percentage / Mean**	**SE / SD**
<20	—	5.2	0.5
20–24	—	20.3	0.9
25–29	—	28.4	1.0
30–34	—	24.1	0.9
35+	—	22.0	0.9
Maternal education level			
No education	—	22.7	1.2
Primary	—	21.6	1.0
Secondary	—	47.2	1.3
Higher	—	12.8	0.9
Currently employed	—	72.3	1.1

Characteristic	n (weighted)	Percentage / Mean	SE / SD
Mean antenatal care visits	—	5.8	±2.1
Four or more ANC visits	—	68.4	1.2
Facility delivery	—	83.6	0.9
Skilled birth attendance	—	81.2	1.0
Any media exposure (at least weekly)	—	76.4	1.1
Distance to facility a big problem	—	24.7	1.1
**HOUSEHOLD CHARACTERISTICS**			
Residence			
Urban	—	48.6	1.5
Rural	—	51.4	1.5
Wealth quintile			

Characteristic	n (weighted)	Percentage / Mean	SE / SD
Poorest	801	23.1	1.4
Poorer	743	21.5	1.2
Middle	697	20.1	1.1
Richer	632	18.2	1.0
Richest	591	17.1	1.3
**CHILD CHARACTERISTICS**			
Child age (months), mean ± SD	—	22.4	±6.8
Child age groups (months)			
12–17	—	30.2	1.0
18–23	—	33.5	1.1
24–35	—	36.3	1.1

Characteristic	n (weighted)	Percentage / Mean	SE / SD
Child sex			
Male	—	50.7	1.0
Female	—	49.3	1.0
Birth order			
First	—	32.1	1.1
Second	—	26.3	0.9
Third	—	17.8	0.8
Fourth or higher	—	23.8	1.0
Vaccination card possession			
Card seen by interviewer	2,815	82.0	0.9
Card reported but not seen	111	3.2	0.5

Characteristic	n (weighted)	Percentage / Mean	SE / SD
No card	64	1.9	0.3

Note: *Percentages may not sum to 100.0 due to rounding. All estimates adjusted for complex survey design with clustering (613 clusters) and stratification (32 strata). SE = Standard Error; SD = Standard Deviation; ANC = Antenatal Care. Sample weights applied to generate nationally representative estimates.*

Healthcare utilization patterns revealed strong engagement with formal services. Mean antenatal care visits reached 5.8, with 68.4% of mothers attending four or more visits as recommended. Facility-based delivery occurred for 83.6% of births, while skilled birth attendants assisted 81.2% of deliveries. Media exposure was widespread, with 76.4% of mothers reporting at least weekly exposure to radio, television, or newspapers. Vaccination card possession rates reached 82.0% with cards physically verified by interviewers.

Immunization Coverage and Vaccination Status

Individual vaccine coverage demonstrated substantial but incomplete uptake across the schedule. BCG coverage reached 92.4%, representing the highest single-vaccine rate. The pentavalent series showed progressive decline from 92.3% for first dose to 88.1% for second dose and 81.9% for third dose. Pneumococcal conjugate vaccine followed similar patterns, achieving 92.0%, 88.0%, and 81.7% for first, second, and third doses respectively. Measles-rubella vaccine showed 79.5% coverage for first dose but dropped dramatically to 42.1% for second dose at 18 months.

Table 2: Individual Vaccine Coverage Rates

Weighted coverage estimates for individual vaccines among children aged 12-35 months, Ghana Demographic and Health Survey 2022

Vaccine	Recommended Schedule	Coverage (%)	SE	95% CI
**TRADITIONAL EPI VACCINES**				
BCG	At birth	92.4	0.6	91.2–93.6
Pentavalent 1	6 weeks	92.3	0.6	91.1–93.5
Pentavalent 2	10 weeks	88.1	0.7	86.7–89.5
Pentavalent 3	14 weeks	81.9	0.9	80.1–83.7
Measles-Rubella 1	9 months	79.5	0.9	77.7–81.3
Measles-Rubella 2	18 months	42.1	1.2	39.7–44.5
**NEWER VACCINES**				
PCV 1	6 weeks	92.0	0.6	90.8–93.2

Vaccine	Recommended Schedule	Coverage (%)	SE	95% CI
PCV 2	10 weeks	88.0	0.7	86.6–89.4
PCV 3	14 weeks	81.7	0.9	79.9–83.5
Rotavirus 1	6 weeks	91.1	0.6	89.9–92.3
Rotavirus 2	10 weeks	85.9	0.8	84.3–87.5
Yellow Fever	9 months	92.3	0.6	91.1–93.5

Note: *Coverage based on vaccination card documentation or maternal recall when cards unavailable. EPI = Expanded Programme on Immunization; PCV = Pneumococcal Conjugate Vaccine; SE = Standard Error; CI = Confidence Interval. All estimates adjusted for complex survey design.*

Composite immunization indicators revealed concerning gaps in schedule completion. Full immunization coverage reached 63.9%, indicating more than one-third of children failed to receive the complete recommended package. Partial immunization affected 29.3% of children who received some but not all vaccines. Zero-dose children, having received no vaccines whatsoever, comprised 4.9% of the population. Vaccination card possession strongly associated with coverage, with 71.2% of children with verified cards achieving full immunization compared to 38.6% of those without cards.

Table 3: Composite Immunization Coverage Indicators

Weighted estimates of full, basic, partial, and zero-dose immunization coverage among children aged 12-35 months, Ghana DHS 2022

Indicator	Definition	Coverage (%)	SE	95% CI
**Full Immunization**	BCG, 3 doses pentavalent, 3 doses PCV, 2 doses rotavirus, MR1, and Y[illegible]	63.9	1.1	61.7–66.1
**Basic Immunization**	Traditional EPI vaccines only: BCG, Penta3, MR1	65.7	1.1	63.5–67.9
**Partial Immunization**	Received at least one vaccine but not fully immunized	31.2	1.0	29.2–33.2
**Zero-Dose Children**	No vaccines received	4.9	0.5	3.9–5.9

Note: *EPI = Expanded Programme on Immunization; PCV = Pneumococcal Conjugate Vaccine; MR1 = Measles-Rubella first dose; YF = Yellow Fever; SE = Standard Error; CI = Confidence Interval. Full immunization represents children who have received all vaccines recommended in the Ghana immunization schedule. Categories are mutually exclusive.*

Dropout Patterns and Timeliness

Dropout analysis identified critical retention challenges. The pentavalent first to third dose dropout reached 10.4%, marginally exceeding the WHO acceptable threshold of 10%. BCG to measles-rubella first dose dropout was 15.0%, indicating substantial attrition through the first year. The most dramatic dropout occurred between measles-rubella first and second doses at 37.5%, suggesting more than one-third of children who received the first dose at nine months failed to return for the second dose at 18 months.

Table 4: Dropout Rates Across Vaccination Schedule

Weighted dropout rates between vaccine doses indicating retention challenges across the immunization schedule, Ghana DHS 2022

Dropout Indicator	Calculation Method	Dropout Rate (%)	SE	95% CI
**WITHIN-SERIES DROPOUT**				
BCG to Penta1	(BCG − Penta1) / BCG × 100	2.6	0.4	1.8–3.4
Penta1 to Penta2	(Penta1 − Penta2) / Penta1 × 100	4.2	0.5	3.2–5.2

Dropout Indicator	Calculation Method	Dropout Rate (%)	SE	95% CI
Penta2 to Penta3	(Penta2 − Penta3) / Penta2 × 100	6.2	0.6	5.0–7.4
**Penta1 to Penta3**	**(Penta1 − Penta3) / Penta1 × 100**	**10.4**	**0.7**	**9.0–11.8**
PCV1 to PCV3	(PCV1 − PCV3) / PCV1 × 100	10.4	0.7	9.0–11.8
Rota1 to Rota2	(Rota1 − Rota2) / Rota1 × 100	5.2	0.6	4.0–6.4
**SCHEDULE-LEVEL DROPOUT**				
Penta3 to Measles1	(Penta3 − MR1) / Penta3 × 100	8.1	0.7	6.7–9.5
**BCG to Measles1**	**(BCG − MR1) / BCG × 100**	**15.0**	**0.9**	**13.2–16.8**

Dropout Indicator	Calculation Method	Dropout Rate (%)	SE	95% CI
**Measles1 to Measles2**	**(MR1 − MR2) / MR1 × 100**	**37.5**	**1.2**	**35.1[illegible] 39.9**

Note: *Dropout rates calculated as proportion of children receiving earlier vaccine who fail to receive later vaccine. Penta = Pentavalent; PCV = Pneumococcal Conjugate Vaccine; Rota = Rotavirus; MR = Measles-Rubella; SE = Standard Error; CI = Confidence Interval. WHO acceptable dropout threshold for Penta1-Penta3 is ≤10%.*

Timeliness analysis revealed widespread delays in age-appropriate administration. Among children with complete date information, BCG timeliness within one month of birth reached only 73.4%. Pentavalent first dose showed the highest timeliness at 83.0%, while pentavalent third dose timeliness declined to 69.2%. Measles-rubella first dose timeliness achieved 78.2%. These patterns indicated that while most children eventually received vaccines, timing frequently deviated from optimal schedules.

Table 5: Age-Appropriate Vaccination Coverage and Timing

Timeliness of vaccine administration and measures of central tendency for age at vaccination, Ghana DHS 2022

Vaccine	Recommended Age Window	Timely (%)	SE	Median Age (months)	Mean Age (months)	SD
BCG	≤1 month	73.4	1.4	0.7	1.1	1.6
Penta1	1.0–2.5 months	83.0	0.9	2.0	2.2	1.6
Penta3	3.0–4.5 months	69.2	1.3	4.2	4.8	2.3
Measles1	8.0–11.0 months	78.2	1.1	10.0	10.3	2.3

Note: *Timeliness calculated among children with complete date information available for 73-80% of vaccinated children. Age calculations based on difference between vaccination date and birth date using DHS Century Month Code format. Windows represent recommended age ranges with appropriate grace periods. SE = Standard Error; SD = Standard Deviation.*

Sociodemographic Disparities in Coverage

Substantial disparities emerged across multiple sociodemographic dimensions. Regional variations proved most striking, with full immunization coverage ranging from 76.4% in Volta Region to 44.4% in Ahafo Region, yielding a 32.0 percentage point gap. These patterns did not follow simple urban-rural or north-south divides, suggesting complex interactions of health system capacity, cultural factors, and local implementation effectiveness.

Table 6: Regional Variations in Full Immunization Coverage

Weighted full immunization coverage estimates by region and ecological zone, ranked from highest to lowest performing, Ghana DHS 2022

Region	Ecological Zone	n	Coverage (%)	SE	95% CI
Volta	Forest	189	76.4	3.9	68.6–84.2
Brong Ahafo	Middle Belt	232	74.1	3.5	67.1–81.1
Greater Accra	Forest	342	73.8	2.9	68.0–79.6
Savannah	Savannah	165	73.5	4.2	65.1–81.9
Upper East	Savannah	254	73.8	3.4	67.0–80.6
Ashanti	Middle Belt	498	72.1	2.5	67.1–77.1
Oti	Forest	178	70.8	4.0	62.8–78.8
Upper West	Savannah	212	69.3	3.8	61.7–76.9
Western North	Coastal	134	68.7	4.7	59.3–78.1

Region	Ecological Zone	n	Coverage (%)	SE	95% CI
Eastern	Forest	287	68.4	3.3	61.8–75.0
Northern	Savannah	326	68.1	3.1	61.9–74.3
Central	Coastal	245	67.5	3.5	60.5–74.5
Western	Coastal	198	60.2	4.2	51.8–68.6
Bono East	Middle Belt	167	59.8	4.5	50.8–68.8
North East	Savannah	189	59.1	4.3	50.5–67.7
Ahafo	Middle Belt	171	44.4	4.5	35.4–53.4
**NATIONAL AVERAGE**					
**Ghana**		**3,787**	**63.9**	**1.1**	**61.7–66.1**

Note: *Regional gap (highest minus lowest) = 32.0 percentage points. Ecological zones: Coastal (Western, Central, Western North); Forest (Greater Accra, Volta, Eastern, Oti); Middle Belt (Ashanti, Brong Ahafo, Ahafo, Bono East); Savannah (Northern, Upper East, Upper West, North East, Savannah). SE = Standard Error; CI = Confidence Interval.*

Wealth-related disparities showed clear gradients. Full immunization coverage increased progressively from 56.3% in the poorest quintile to 73.7% in the richest quintile, creating a 17.4 percentage point gap. Educational disparities paralleled wealth patterns in magnitude, with coverage increasing from 55.6% among children of mothers without education to 72.8% for mothers with higher education, yielding a 17.2 percentage point gap. Urban areas demonstrated 66.9% coverage compared to 61.1% in rural areas.

Table 7: Full Immunization Coverage by Sociodemographic Characteristics

Weighted full immunization coverage estimates stratified by key sociodemographic variables, Ghana DHS 2022

Characteristic	n	Coverage (%)	SE	95% CI	P-value
**OVERALL**					
**Total**	3,787	63.9	1.1	61.7–66.1	—
**RESIDENCE**					
Urban	1,795	66.9	1.5	63.9–69.9	<0.001
Rural	1,992	61.1	1.5	58.1–64.1	

Characteristic	n	Coverage (%)	SE	95% CI	P-value
**WEALTH QUINTILE**					
Poorest	801	56.3	2.0	52.3–60.3	<0.001
Poorer	743	62.6	2.0	58.6–66.6	
Middle	697	63.3	2.5	58.3–68.3	
Richer	632	66.4	2.7	61.0–71.8	
Richest	591	73.7	2.9	67.9–79.5	
**MATERNAL EDUCATION**					
No education	897	55.6	2.0	51.6–59.6	<0.001
Primary	768	63.2	2.1	59.0–67.4	
Secondary	1,812	66.2	1.4	63.4–69.0	
Higher	455	72.8	2.5	67.8–77.8	

Characteristic	n	Coverage (%)	SE	95% CI	P-value
**CHILD SEX**					
Male	1,942	63.6	1.4	60.8–66.4	0.721
Female	1,845	64.2	1.5	61.2–67.2	
**BIRTH ORDER**					
First	1,216	67.1	1.7	63.7–70.5	<0.001
Second	996	65.3	1.9	61.5–69.1	
Third	674	62.4	2.3	57.8–67.0	
Fourth or higher	901	59.8	2.0	55.8–63.8	
**ANTENATAL CARE**					
Four or more visits	2,591	68.9	1.2	66.5–71.3	<0.001
Fewer than four visits	1,196	53.7	1.9	49.9–57.5	

Characteristic	n	Coverage (%)	SE	95% CI	P-value
**PLACE OF DELIVERY**					
Facility delivery	3,167	66.1	1.1	63.9–68.3	<0.001
Home delivery	620	48.3	2.5	43.3–53.3	
**MEDIA EXPOSURE**					
Any media exposure	2,893	66.2	1.2	63.8–68.6	<0.001
No media exposure	894	55.8	2.1	51.6–60.0	

Note: *P-values from design-adjusted chi-square tests. SE = Standard Error; CI = Confidence Interval. All estimates account for complex survey design with clustering and stratification.*

Healthcare utilization patterns showed strong associations with immunization outcomes. Mothers with four or more antenatal visits achieved 68.9% full immunization compared to 53.7% among those with fewer visits. Facility delivery associated with 66.1% coverage versus 48.3% for home delivery. Media exposure correlated with 66.2% coverage compared to 55.8% without exposure.

Predictors of Full Immunization

Multivariate logistic regression revealed independent predictors after controlling for confounding. Wealth remained significant, with children in the rich tertile showing 43% higher odds of full

immunization compared to the poor tertile. Maternal secondary or higher education associated with 35% higher odds relative to primary or less education.

Table 8: Predictors of Full Immunization Coverage: Multivariate Logistic Regression Analysis

Survey-weighted adjusted odds ratios from progressive multivariate logistic regression models, Ghana DHS 2022

Predictor	Model 1 aOR (95% CI)	Model 2 aOR (95% CI)	Model 3 aOR (95% CI)	P-value (Model 3)
**SOCIODEMOGRAPHIC FACTORS**				
**Residence**				
Rural (ref)	1.00	1.00	1.00	—
Urban	1.32 (1.03–1.69)	1.21 (0.94–1.56)	1.18 (0.91–1.53)	0.214
**Wealth Tertile**				
Poor (ref)	1.00	1.00	1.00	—

Predictor	Model 1 aOR (95% CI)	Model 2 aOR (95% CI)	Model 3 aOR (95% CI)	P-value (Model 3)
Middle	1.23 (0.98–1.54)	1.16 (0.92–1.46)	1.14 (0.90–1.44)	0.278
Rich	1.68 (1.27–2.22)	1.47 (1.10–1.96)	1.43 (1.07–1.91)	0.016
**Maternal Education**				
Primary or less (ref)	1.00	1.00	1.00	—
Secondary or higher	1.54 (1.21–1.96)	1.38 (1.08–1.77)	1.35 (1.05–1.73)	0.019
**Child Sex**				
Female (ref)	1.00	1.00	1.00	—
Male	0.97 (0.81–1.16)	0.98 (0.82–1.17)	0.98 (0.82–1.17)	0.809

Predictor	Model 1 aOR (95% CI)	Model 2 aOR (95% CI)	Model 3 aOR (95% CI)	P-value (Model 3)
**Birth Order**				
First (ref)	1.00	1.00	1.00	—
Second	0.92 (0.74–1.15)	0.93 (0.74–1.16)	0.94 (0.75–1.18)	0.589
Third	0.81 (0.63–1.04)	0.84 (0.65–1.09)	0.85 (0.65–1.10)	0.214
Fourth or higher	0.72 (0.56–0.93)	0.76 (0.58–0.99)	0.77 (0.59–1.01)	0.057
**HEALTHCARE UTILIZATION**				
**Antenatal Care**				
<4 visits (ref)	—	1.00	1.00	—

Predictor	Model 1 aOR (95% CI)	Model 2 aOR (95% CI)	Model 3 aOR (95% CI)	P-value (Model 3)
4+ visits	—	1.67 (1.29–2.17)	1.64 (1.26–2.13)	<0.001
**Place of Delivery**				
Home (ref)	—	1.00	1.00	—
Facility	—	1.89 (1.43–2.49)	1.81 (1.37–2.40)	<0.001
**INFORMATION ACCESS**				
**Media Exposure**				
No exposure (ref)	—	—	1.00	—
Any exposure	—	—	1.37 (1.09–1.73)	0.007

Predictor	Model 1 aOR (95% CI)	Model 2 aOR (95% CI)	Model 3 aOR (95% CI)	P-value (Model 3)
**Distance Barrier**				
Not a problem (ref)	—	—	1.00	—
Big problem	—	—	0.78 (0.63–0.97)	0.024
**MODEL FIT STATISTICS**				
AIC	4,183	4,121	4,108	—
Sample size	3,787	3,787	3,787	—

Note: *Model 1: Sociodemographic factors only. Model 2: Adds healthcare utilization. Model 3 (Full model): Adds information access and distance barriers. aOR = adjusted Odds Ratio; CI = Confidence Interval; AIC = Akaike Information Criterion; ref = reference category. All models adjusted for complex survey design with clustering and stratification. P-values <0.05 considered statistically significant.*

Healthcare utilization variables demonstrated particularly strong independent effects. Four or more antenatal care visits associated with 64% higher odds of full immunization. Facility delivery showed 81% higher odds compared to home delivery. Media exposure associated with 37% higher odds, while reporting distance as a big problem correlated with 22% lower odds.

Stratified analysis by residence revealed differential patterns. In urban areas, wealth effects were attenuated and non-significant, while healthcare utilization maintained strong associations. Rural areas demonstrated stronger socioeconomic gradients, with the rich tertile showing 82% higher odds and facility delivery demonstrating 108% higher odds.

Equity Analysis

The concentration index of 0.0607 indicated modest pro-rich inequality. The equity ratio of 1.31 showed children in the richest quintile had 1.31 times the coverage of those in the poorest quintile. Regional inequality substantially exceeded socioeconomic dimensions at 32.0 percentage points between highest and lowest performing regions, compared to 17.4 for wealth and 17.2 for education.

Intersectional analysis identified compounding disadvantages. The most advantaged group achieved 78.3% coverage, while the most disadvantaged demonstrated 59.4% coverage, yielding an 18.9 percentage point intersectional gap.

Table 9: Equity Analysis: Multiple Measures of Immunization Inequality

Comprehensive equity assessment using complementary inequality measures, Ghana DHS 2022

Equity Measure	Value	Interpretation
**WEALTH-RELATED INEQUALITY**		
Concentration Index (Kakwani formula)	0.0607	Pro-rich inequality; positive value indicates higher coverage among wealthier households
Rate Ratio (Richest/Poorest)	1.31	Richest quintile has 1.31 times the coverage of poorest quintile
Absolute Gap (Richest − Poorest)	17.4 pp	17.4 percentage point difference between richest and poorest quintiles
Slope Index of Inequality	0.81	Difference in log-odds of immunization between extremes of wealth distribution
Relative Index of Inequality	2.25	Top of wealth distribution has 2.25 times the odds of bottom
**EDUCATIONAL INEQUALITY**		
Absolute Gap (Higher − None)	17.2 pp	17.2 percentage point difference between mothers with higher education and no education

Equity Measure	Value	Interpretation
Rate Ratio (Higher/None)	1.31	Children of mothers with higher education have 1.31 times coverage of those with no education
**GEOGRAPHIC INEQUALITY**		
Urban-Rural Gap	5.8 pp	Urban areas have 5.8 percentage points higher coverage than rural areas (urban advantage)
Regional Gap (Highest − Lowest)	32.0 pp	32.0 percentage point difference between best (Volta: 76.4%) and worst (Ahafo: 44.4%) regions
**INTERSECTIONAL INEQUALITY**		
Intersectional Gap	18.9 pp	18.9 percentage point difference between most advantaged (urban, rich, educated) and most disadvantaged (rural, poor, no education) groups
**INTERPRETATION SUMMARY**		
All inequality measures indicate **pro-rich, pro-educated, pro-urban patterns** with **regional inequality exceeding socioeconomic inequality**. The concentration index of 0.0607		

Equity Measure	Value	Interpretation
		represents **modest but significant pro-rich inequality**. The largest inequality dimension is **geographic/regional** (32.0 pp), followed by **wealth** (17.4 pp) and **education** (17.2 pp). Urban-rural gap is smallest (5.8 pp), suggesting that within-setting variations matter more than simple urban-rural divide.

Note: *pp = percentage points. Concentration index ranges from -1 (extreme pro-poor) to +1 (extreme pro-rich), with 0 indicating perfect equality. Rate ratios >1 indicate advantage for the first group. All measures based on survey-weighted estimates adjusted for complex design.*

Policy Implementation Effectiveness

Comparison against national targets revealed mixed performance. Only yellow fever vaccine exceeded the 90% target. Full immunization coverage of 63.9% achieved only 71% of the 90% target, falling 26.1 percentage points short. The 37.5% dropout between measles-rubella first and second doses dramatically exceeded any reasonable benchmark, representing a critical programme weakness requiring urgent attention.

Table 10: Policy Implementation Effectiveness and Target Achievement

Comparison of actual coverage against national and international targets for key immunization indicators, Ghana DHS 2022

Indicator	Target (%)	Actual (%)	SE	Gap (pp)	% of Target	Target Status
**PRIMARY TARGETS**						
Full Immunization	90.0	63.9	1.1	−26.1	71.0	Not achieved
BCG	95.0	92.4	0.6	−2.6	97.3	Near target
Penta3	90.0	81.9	0.9	−8.1	91.0	Near target
Measles1	90.0	79.5	0.9	−10.5	88.3	Not achieved
**NEWER VACCINE TARGETS**						
PCV3	90.0	81.7	0.9	−8.3	90.8	Near target
Rotavirus 2	90.0	85.9	0.8	−4.1	95.4	Near target
Yellow Fever	90.0	92.3	0.6	+2.3	102.6	**Achieved**
**DROPOUT BENCHMARKS**						

Indicator	Target (%)	Actual (%)	SE	Gap (pp)	% of Target	Target Status
Penta1-Penta3 Dropout	≤10.0	10.4	0.7	+0.4	—	Marginally exceeded
BCG-Measles1 Dropout	≤10.0	15.0	0.9	+5.0	—	**Exceeded**
**SUMMARY ASSESSMENT**						
**Targets Achieved:** 1 of 6 primary coverage targets (Yellow Fever only) **Near Target (≥88%):** BCG, Penta3, PCV3, Rotavirus 2 **Below Target:** Full immunization, Measles1 **Dropout Performance:** Penta1-Penta3 marginally exceeds acceptable threshold; BCG-Measles1 substantially exceeds threshold						

Note: *Targets based on Ghana EPI Comprehensive Multi-Year Plan 2021-2025 and WHO/UNICEF recommendations. pp = percentage points; SE = Standard Error. Near target defined as achieving ≥88% of target. Dropout benchmarks based on WHO acceptable thresholds for programme quality.*

Discussion

Main Findings and Public Health Significance

This analysis of Ghana's childhood immunization programme reveals achievements in establishing broad access alongside persistent challenges in completion, equity, and timeliness. The 63.9% full immunization coverage represents substantial progress from 57% reported in the 2014 survey, yet indicates more than one-third of Ghanaian children fail to receive complete recommended protection. The low zero-dose prevalence of 4.9% demonstrates that initial contact with immunization services reaches the vast majority of children, suggesting access barriers are not the primary driver of incomplete coverage. Instead, the substantial partial immunization rate of 29.3% and elevated dropout rates point to retention and schedule completion as dominant programme weaknesses.

The programme's critical challenge lies in maintaining engagement throughout the complete schedule rather than achieving initial access. This distinction has important programmatic implications, as retention interventions differ fundamentally from access-focused strategies. The dramatic 37.5% dropout between measles-rubella first and second doses represents a particularly severe retention failure, with more than one-third of children who successfully received the nine-month dose failing to return for the 18-month dose. This pattern suggests systematic weaknesses in tracking children, maintaining parental awareness of second-year vaccines, and ensuring convenient service delivery for toddlers beyond infancy.

Equity Patterns and System-Level Determinants

The substantial inequalities documented across wealth, education, and geography indicate immunization programme benefits distribute unevenly despite universal policy frameworks. The

concentration index of 0.061 represents modest pro-rich inequality relative to many other health outcomes, reflecting some success of free immunization policy and routine service delivery in mitigating economic barriers. However, the persistence of 17.4 percentage point wealth gaps indicates incomplete success in achieving equity goals. The parallel magnitude of educational disparities suggests maternal knowledge, health literacy, and empowerment substantially influence immunization outcomes beyond household resources alone.

Regional disparities exceeding socioeconomic inequalities in magnitude highlight health system capacity variations as critical determinants of coverage. The fact that regional gaps surpass household-level wealth gaps suggests that where families live matters as much or more than their individual resources. This pattern indicates implementation effectiveness, health workforce distribution, supervision quality, cold chain functionality, and local cultural factors create geographic variations that household resources cannot fully overcome. The lack of simple urban-rural or north-south patterns further emphasizes the complexity of these system-level determinants.

The finding that urban-rural gaps were relatively modest while regional variations were substantial challenges simplistic narratives about rural disadvantage. Some remote regions achieved coverage exceeding more accessible areas, while some predominantly urban regions underperformed relative to their infrastructure advantages. These patterns suggest that programmatic effectiveness depends more on implementation quality, supervision, and demand generation than simple physical access. Successful rural regions likely employed effective outreach strategies, maintained functional supply chains despite logistical challenges, and built strong community engagement.

Healthcare Continuity and Family-Level Determinants

The strong associations between immunization completion and broader healthcare engagement patterns reveal that vaccination coverage reflects family-level orientations toward formal health services extending well beyond immunization programmes. The adjusted odds ratios of 1.64 for four or more antenatal visits and 1.81 for facility delivery, persisting after controlling for socioeconomic factors, suggest these associations represent genuine behavioral patterns rather than merely socioeconomic confounding. Families engaging across the maternal and child health continuum demonstrate consistently better outcomes, indicating shared determinants of healthcare utilization.

These patterns suggest multiple potential mechanisms. Healthcare engagement may reflect underlying health orientation, with families prioritizing preventive care consistently utilizing services across domains. Trust in health services, built through positive antenatal and delivery experiences, may facilitate subsequent immunization adherence. Practical factors including understanding clinic procedures, relationships with healthcare workers, and familiarity with navigating health facilities may reduce barriers to immunization visits.

The attenuation of wealth and education associations when healthcare utilization variables were added to regression models suggests healthcare engagement partially mediates socioeconomic effects on immunization. Wealthier and more educated mothers may achieve higher immunization coverage partly through greater healthcare utilization, indicating that interventions improving healthcare access and continuity could reduce socioeconomic disparities. However, the persistence of significant wealth and education effects after healthcare utilization adjustment suggests additional pathways including health literacy and healthcare provider communication patterns.

Timeliness and Schedule Adherence

The timeliness findings reveal an under-recognized dimension of programme quality with important implications for disease protection. While coverage eventually reaches relatively high levels for most vaccines, substantial proportions occur beyond recommended schedules, leaving children vulnerable during critical periods. Only 73% of BCG vaccinations within one month of birth and 69% of pentavalent third dose within appropriate windows indicate widespread delays. These patterns suggest healthcare scheduling systems, reminder mechanisms, and counseling about optimal timing require strengthening.

The relatively high timeliness for pentavalent first dose compared to third dose indicates initial postnatal contact often occurs appropriately while subsequent visits show increasing delays. This pattern points to retention and recall as primary challenges rather than initial access barriers. Interventions could include structured scheduling systems at the time of each visit, reminder mechanisms for upcoming visits, and community-level tracking ensuring eligible children receive timely vaccines.

Comparison with Regional and Global Evidence

These findings align with existing literature from Ghana and similar contexts. The 63.9% full immunization coverage falls between previous estimates, confirming gradual but incomplete progress. [11,13-14] This performance places Ghana in the middle range among sub-Saharan African nations, exceeding fragile states but trailing southern African countries with more developed health infrastructure. [5,15] The concentration index of 0.061 falls in the lower range of inequalities documented across African nations, reflecting Ghana's relatively developed health system while indicating room for substantial improvement. [5,16]

The 37.5% dropout between measles-rubella first and second doses exceeds rates documented in most comparable settings. Studies from Nigeria, Uganda, and Malawi report measles second dose dropout of 20 to 40%, with explanations centering on reduced perceived urgency after first year completion, competing parental demands as children age, and health system failures in tracking eligible children. [17,18]

Implications for Policy and Practice

These findings generate clear priorities for policy and programme strengthening. The elevated dropout rates demand urgent intervention through robust tracking systems identifying children overdue for vaccines, structured follow-up mechanisms including home visits or phone reminders, and intensified community mobilization targeting second-year vaccines. Several African countries have demonstrated successful dropout reduction through SMS reminder systems and community dialogue approaches. [19-21]

The strong associations between immunization and maternal healthcare engagement suggest enhanced integration across the maternal and child health continuum could improve outcomes. Structured immunization counseling during antenatal care could leverage existing pregnancy contact to improve subsequent vaccination adherence. Addressing documented inequalities requires moving beyond universal strategies toward targeted interventions reaching disadvantaged populations. Regional variations suggest differentiated approaches adapted to local contexts would improve effectiveness, with high-performing regions serving as models for peer learning.

Strengths and Limitations

This study benefits from nationally representative data with rigorous sampling enabling robust inference and adequate power for subgroup analyses. Multiple complementary analytical

approaches provided convergent evidence on multidimensional inequality. Several limitations warrant acknowledgment. The cross-sectional design prevents causal inference despite strong observed associations, and unmeasured confounding remains possible. Maternal recall introduces potential reporting bias. Generalizability beyond Ghana requires caution given its political stability and relatively developed health infrastructure.

Future Research Directions

Several research priorities emerge from this analysis. Longitudinal cohort studies would enable stronger causal inference. Implementation research could identify scalable best practices. Investigation of zero-dose children requires intensified focus given their concentration in disadvantaged populations.

Conclusions

Ghana's childhood immunization programme has achieved substantial progress in establishing broad access to vaccines, with the vast majority of children receiving at least some recommended vaccines. However, critical weaknesses in retention through the complete schedule, timeliness of administration, and equitable distribution constrain progress toward universal coverage goals. Zero-dose prevalence remains below five percent while partial immunization affects nearly thirty percent, indicating the primary challenge has shifted from initial access to completion and retention. The 37.5 percent dropout between measles-rubella first and second doses represents an urgent programme crisis requiring immediate attention through enhanced tracking, reminder systems, and community mobilization targeting second-year vaccines. Regional disparities exceeding socioeconomic inequalities highlight health system capacity variations as critical determinants. Priority policy actions should include implementing robust defaulter tracking

systems, integrating immunization counseling throughout antenatal care, and deploying targeted outreach to disadvantaged populations.

Acknowledgments

We acknowledge the Ghana Statistical Service, Ghana Health Service, and ICF International for conducting the 2022 Ghana Demographic and Health Survey and making the data publicly available. We thank the survey field staff who collected the data and the Ghanaian families who participated in the survey. We appreciate the Demographic and Health Survey Program for providing technical assistance to the survey and maintaining the data access platform.

Author Contributions

Ahlam Tunteeya Saani: Conceptualization, Methodology, Formal analysis, Investigation, Data curation, Writing - original draft, Writing - review and editing

Goldfield Edem Azumah: Methodology, Investigation, Data curation, Supervision, Writing - review and editing

Both authors reviewed and approved the final manuscript for submission, had full access to all data in the study, and accept responsibility for the decision to submit for publication. Ahlam Tunteeya Saani and Goldfield Edem Azumah directly accessed and verified the underlying data reported in the manuscript.

Data Sharing Statement

The deidentified participant data from this study are publicly available through the Demographic and Health Survey Program. The dataset includes individual-level responses from the 2022 Ghana Demographic and Health Survey for children aged twelve to thirty-five months, encompassing immunization records, sociodemographic characteristics, healthcare utilization patterns, and geographic identifiers. The study protocol and statistical analysis plan are available from the

corresponding author upon reasonable request. Data became available in March 2023 and remain permanently accessible at https://dhsprogram.com. Researchers can access the data by registering for a free account and submitting a brief research proposal. The DHS Program typically approves requests within one to two business days, requiring only standard data use agreements ensuring ethical use and participant confidentiality protection. Data for this analysis were accessed and downloaded from the DHS Program on 8th October, 2025.

Funding Statement

The authors received no specific funding for this work.

Declaration of interests

The authors declare that they have no competing interests related to this work. No author has any financial relationship with commercial entities that might have an interest in the submitted work. No author has any relationships or activities that readers could perceive to have influenced the submitted work.

References

1. Alanazi FT, Alharbi BN, Aljuaid TH, Alammar FA, Almarzouq YF, Albalawi I, Almutairi MN, Alshaghroud SM, Alhaosawi ME, Albalawi MA, Alorf AA. The impact of vaccinations on disease prevention: A comprehensive analysis of their role in enhancing global public health and reducing morbidity and mortality rates. International journal of health sciences. 2024;8(S1):1885-907.

2. Wallace AS, Ryman TK, Privor-Dumm L, Morgan C, Fields R, Garcia C, Sodha SV, Lindstrand A, Nic Lochlainn LM. Leaving no one behind: Defining and implementing an integrated life course approach to vaccination across the next decade as part of the immunization Agenda 2030. Vaccine. 2024 Apr 8;42:S54-63.

3. Lindstrand A, Cherian T, Chang-Blanc D, Feikin D, O'Brien KL. The world of immunization: achievements, challenges, and strategic vision for the next decade. The Journal of Infectious Diseases. 2021 Oct 1;224(Supplement_4):S452-67.

4. Myemba DT, Smets L, Sunguya BF, Vandaele N, Decouttere C. Challenges and strategies for sustainable and resilient immunization systems in sub-Saharan Africa: A comprehensive scoping review. Vaccine. 2025 Jan 25;45:126639.

5. Bobo FT, Asante A, Woldie M, Dawson A, Hayen A. Child vaccination in sub-Saharan Africa: Increasing coverage addresses inequalities. Vaccine. 2022 Jan 3;40(1):141-50.

6. Gebeyehu NA, Asmare Adela G, Dagnaw Tegegne K, Birhan Assfaw B. Vaccination dropout among children in Sub-Saharan Africa: Systematic review and meta-analysis. Human Vaccines & Immunotherapeutics. 2022 Dec 30;18(7):2145821.

7. Walana W, Al-Azab M, Yabasin IB, Abdul-Mumin A. Childhood immunization in Ghana: Tracing the history and projecting the future. Public Health Challenges. 2024 Jun;3(2):e176.

8. Muhoza P, Shah MP, Gao H, Amponsa-Achiano K, Quaye P, Opare W, Okae C, Aboyinga PN, Opare KL, Wardle MT, Wallace AS. Predictors for Uptake of Vaccines Offered during the Second Year of Life: Second Dose of Measles-Containing Vaccine and Meningococcal Serogroup A-Containing Vaccine, Ghana, 2020. Vaccines. 2023 Sep 23;11(10):1515.

9. Sem S, Sarfo JO, Amoadu M, Hormenu T. Uptake and determinants of routine vaccines among children aged 12–23 months in adansi South district of Ghana. a cross-sectional study. BMC pediatrics. 2025 Mar 17;25(1):198.

10. Danso SE, Frimpong A, Seneadza NA, Ofori MF. Knowledge, attitudes, and practices of caregivers on childhood immunization in Okaikoi sub-metro of Accra, Ghana. Frontiers in Public Health. 2023 Sep 15;11:1230492.

11. Tekeba B, Tamir TT, Zegeye AF. Prevalence and determinants of full vaccination coverage according to the national schedule among children aged 12–35 months in Ghana. Scientific Reports. 2025 Jan 2;15(1):13.

12. Akanpaabadai EA, Adiak AA, Nukpezah RN, Adokiya MN, Adjei SE, Boah M. Population-based cross-sectional study of factors influencing full vaccination status of children aged 12-23 months in a rural district of the Upper East Region, Ghana. BMC pediatrics. 2024 Mar 8;24(1):168.

13. Budu E, Darteh EK, Ahinkorah BO, Seidu AA, Dickson KS. Trend and determinants of complete vaccination coverage among children aged 12-23 months in Ghana: analysis of data from the 1998 to 2014 Ghana demographic and health surveys. Plos one. 2020 Oct 1;15(10):e0239754.

14. Xavier SP, Mahoche M, Rondó PH, da Silva AM, Flores-Ortiz R, Victor A. Addressing inequalities in vaccination coverage among children aged 12 to 23 months in ten Sub-Saharan African countries: Insights from DHS and MIS Data (2017-2022). MedRxiv. 2024 Dec 14:2024-12.

15. Fenta SM, Biresaw HB, Fentaw KD, Gebremichael SG. Determinants of full childhood immunization among children aged 12–23 months in sub-Saharan Africa: a multilevel analysis using Demographic and Health Survey Data. Tropical medicine and health. 2021 Apr 1;49(1):29.

16. Gasana J, Niyoyita JC, Bigari JD, Sindikubwabo I, Muhimpundu MA, Sibomana H, Mushinge D, Nsanzabaganwa C, Musanabaganwa C, Mutesa L. Immunization Saves Lives: Maintaining Good Immunization Coverage To Prevent Potential Outbreaks. Rwanda Public Health Bulletin. 2023;4(4):11-5

17. Gebeyehu NA, Asmare Adela G, Dagnaw Tegegne K, Birhan Assfaw B. Vaccination dropout among children in Sub-Saharan Africa: Systematic review and meta-analysis. Human Vaccines & Immunotherapeutics. 2022 Dec 30;18(7):2145821. .

18. Okoro OO, Anoke CI, Olaiya PA, Ogbu MA, Okoro OG. Understanding the Socio-Economic and Demographic Factors Affecting Uptake of Second Dose Measles Vaccination (MCV2) among Children Under 5 in Ebonyi State, South-Eastern Nigeria. Texila International Journal of Academic Research. 2024:1-4.

19. Obi-Jeff C, Garcia C, Onuoha O, Adewumi F, David W, Bamiduro T, Aliyu AB, Labrique A, Wonodi C. Designing an SMS reminder intervention to improve vaccination uptake in Northern Nigeria: a qualitative study. BMC health services research. 2021 Aug 20;21(1):844.

20. Ehlman DC, Magoola J, Tanifum P, Wallace AS, Behumbiize P, Mayanja R, Luzze H, Yukich J, Daniels D, Mugenyi K, Baryarama F. Evaluating a Mobile Phone–Delivered Text Message Reminder Intervention to Reduce Infant Vaccination Dropout in Arua, Uganda:

Protocol for a Randomized Controlled Trial. JMIR Research Protocols. 2021 Feb 24;10(2):e17262.

21. Mekonnen ZA, Gelaye KA, Were MC, Tilahun B. Acceptability, barriers and facilitators of mobile text message reminder system implementation in improving child vaccination: a qualitative study in Northwest Ethiopia. Journal of multidisciplinary healthcare. 2021 Mar 9:605-16.

22. Robson J, Bao J, Wang A, McAlister H, Uwizihiwe JP, Sayinzoga F, Sibomana H, Koswin K, Wong J, Zlotkin S. Making sense of Rwanda's remarkable vaccine coverage success. Int J Healthc. 2020;6(1):56.
