# Supplementary material for: Sociodemographic and geographic determinants of childhood immunization coverage and equity in Ghana: Analysis of the 2022 demographic and health survey
Source: PLoS One. 2026 Apr 15;21(4):e0337505. doi: 10.1371/journal.pone.0337505 (PMC13082596; doi:10.1371/journal.pone.0337505)
Supplement: Attachment — Submitted filename: PONE-D-25-59990 - Response to reviewers.docx [file pone.0337505.s003.docx]

**Response to Reviewers**

Manuscript Number: PONE-D-25-59990

Title: Sociodemographic and Geographic Determinants of Childhood Immunization Coverage and Equity in Ghana: Analysis of the 2022 Demographic and Health Survey

Journal: PLOS ONE

Dear Editorial Team,

We thank the Academic Editor and Reviewer 1 for their thorough and constructive evaluation of our manuscript. Their comments have substantially strengthened the paper, particularly regarding the critical methodological issue of measles-rubella second dose (MR2) age eligibility and the need for clearer presentation of analytical decisions. We have carefully addressed each comment and made comprehensive revisions to the manuscript. All changes are tracked in the revised manuscript file.

Below we provide a point-by-point response to each comment. **Editor and reviewer comments are shown in italics**, and our responses follow in regular text. Page and line references correspond to the revised manuscript with tracked changes.

**EDITOR COMMENTS**

**Comment 1: Definition of “coverage plateau”**

*INTRO, line 44: “coverage plateau” could be jargon — suggest defining it for a broad readership.*

**Response:** We agree and have added a parenthetical definition. The revised text now reads: “…with coverage plateaus, periods where immunization rates stagnate despite continued programmatic investments, and recent reversals threatening progress toward Sustainable Development Goal targets.” (Introduction, paragraph 1)

**Comment 2: Measles-rubella second dose (MR2) data presentation**

*Variable definitions: MR second dose — make sure data for MR2 is presented clearly since the primary outcome doesn’t include MR2 but conclusions relate to it. ~30% of subjects are <18 months. Not clear how data for 18-month MR2 were analyzed.*

**Response:** This was critical and well-taken point, also raised repeatedly by Reviewer 1. We have made the following substantive revisions:

(a) We re-analysed the entire dataset, restricting all MR2-related analyses (coverage, dropout) to children aged 18–35 months who were actually eligible for the 18-month dose. MR2 coverage among age-eligible children was 62.1%, compared to 46.8% when all children aged 12–35 months were included as the denominator.

(b) The MR1→MR2 dropout rate was recalculated using only children aged 18–35 months, yielding a corrected estimate of 28.9%—substantially lower than the original 37.5% that erroneously included age-ineligible children in the denominator.

(c) The full immunization definition was revised to exclude MR2, as this vaccine targets 18 months and cannot reasonably be expected among children aged 12–17 months (30.2% of the sample). The revised full immunization estimate is 69.5% (95% CI: 67.3–71.7%), reflecting BCG, three doses of pentavalent and polio vaccines, three doses of pneumococcal conjugate vaccine, two doses of rotavirus vaccine, one dose each of measles-rubella first dose and yellow fever vaccine. All tables and text have been updated accordingly.

(d) Both the unadjusted (37.5%) and age-corrected (28.9%) MR1→MR2 dropout rates are now presented in the Results and Discussion sections, with explicit explanation of why the corrected estimate is more appropriate. (See Results, Dropout Analysis section; Discussion, paragraphs on MR2 dropout)

**Comment 3: How wealth quintiles were collapsed into tertiles**

*Line 133: How were quintiles collapsed into tertiles? (1+2, 3, 4+5? or 1, 2+3+4, 5?)*

**Response:** We have specified the grouping scheme in the Methods section. The revised text now reads: “For multivariate analysis, wealth quintiles were collapsed into tertiles by combining the poorest and poorer quintiles into a ‘poor’ category, retaining the middle quintile as ‘middle,’ and combining the richer and richest quintiles into a ‘rich’ category, to improve statistical power while maintaining meaningful discrimination across wealth groups.” (Methods, Variable Definitions)

**Comment 4: Table 1 percentages do not add up**

*Table 1: Vaccine card possession categories don’t add to anything like 100% (<88%). Maternal education sums to >100%.*

**Response:** Both issues have been corrected:

(a) **Vaccination card possession:** The original Table 1 omitted two categories. We have added “Not available / Don’t know” (4.6%) and “Other / Missing” (8.3%) rows, and all categories now sum to 100.0%. These categories represent children whose card information was not ascertained or where the response was missing from the dataset.

(b) **Maternal education:** The original percentages (22.7 + 21.6 + 47.2 + 12.8 = 104.3%) contained errors from an earlier analysis run. The corrected distribution is: No education 22.7%, Primary 16.0%, Secondary 52.8%, Higher 8.7%. These corrected values have been updated throughout the manuscript.

**Comment 5: “Central tendency” terminology**

*Line 236: What is meant by “central tendency”?*

**Response:** We have replaced the jargon phrase “measures of central tendency” in the Table 5 subtitle with plain language: “Timeliness of vaccine administration and median and mean age at vaccination, Ghana DHS 2022.” (Table 5 title)

**REVIEWER 1 COMMENTS (Kathleen Walker)**

**Comment 1: MR2 age eligibility — children aged 12–17 months are not eligible for MR2**

*The measles vaccination schedule in Ghana administers MR1 at 9 months and MR2 at 18 months. Approximately 30.2% of the study sample is aged 12–17 months and would not yet be eligible for MR2. How was this handled in the analysis? Were dropout calculations restricted to children ≥18 months? This concern affects the interpretation of the 37.5% MR1→MR2 dropout rate and the full immunization estimate.*

**Response:** We appreciate this critical observation, which fundamentally affected our main findings. We have made the following comprehensive revisions:

First, we revised the full immunization definition to exclude MR2, as children aged 12–17 months (30.2% of the sample) cannot reasonably have received this 18-month dose. The revised full immunization coverage is 69.5% (95% CI: 67.3–71.7%).

Second, all MR2-specific analyses are now restricted to children aged 18–35 months (n = 2,644). Among this age-eligible sub-sample, MR2 coverage was 62.1%, and the MR1→MR2 dropout rate was 28.9%—substantially lower than the original 37.5% estimate that included age-ineligible children.

Third, we now present both the unadjusted and age-corrected dropout estimates in the Results and Discussion, explaining why the corrected estimate provides a more accurate programmatic performance measure.

These changes are reflected throughout the Abstract, Results, Discussion, and Conclusions sections, as well as in all relevant tables.

**Comment 2: Proportion receiving both MR1 and MR2**

*What percentage of children received both doses of measles-rubella vaccine?*

**Response:** We have added this information to the Dropout Analysis section. Among children aged 18 to 35 months eligible for both measles-rubella doses, we now report the proportion who received both MR1 and MR2, those who received MR1 only, and those who received neither dose. This provides the complete picture of measles-rubella vaccination status requested by the reviewer. (See Results, Dropout Analysis)

**Comment 3: Urban/rural sample proportions and weighting**

*Approximately 60% of Ghanaians live in urban areas, yet the sample is ~49% urban and ~51% rural. How does the weighting account for this?*

**Response:** We have added an explanation in the Results section clarifying that the sample distribution of 48.6% urban and 51.4% rural reflects the DHS sampling design, which oversamples certain strata to ensure adequate subgroup representation. Survey sampling weights adjust for these design decisions and differential response rates, producing nationally representative estimates that account for Ghana’s actual population distribution, including its approximately 57% urban population per the 2021 Population and Housing Census. All reported estimates in the manuscript are weighted and therefore nationally representative. (See Results, Sample Characteristics)

**Comment 4: Reasons for non-vaccination**

*Were parents asked why children did not receive second-year vaccines? What are the reported barriers?*

**Response:** While the DHS instrument does collect some information on reasons for non-vaccination, these variables capture reasons for non-vaccination generally rather than specifically for second-year vaccines. We have acknowledged this as an important limitation and a direction for future research. The revised Limitations section now states: “Although the DHS collects self-reported reasons for non-vaccination, the present analysis employed a binary indicator of vaccination status and did not examine specific self-reported barriers. Understanding the particular demand-side and supply-side factors driving dropout for second-year vaccines represents an important avenue for future investigation.” (See Limitations)

**Comment 5: Determinants of regional differences**

*Which factors contribute most to regional disparities? Are wealth, education, or other factors driving these gaps?*

**Response:** We have expanded the Discussion section to address this important question more substantively. The revised text now includes a paragraph discussing the relative contributions of health system factors to regional performance differences: “Further investigation is warranted to disentangle the relative contributions of health workforce density, cold chain functionality, supervision quality, and community engagement to regional performance differences. While the present cross-sectional design limits causal attribution, the observation that some resource-constrained regions outperform wealthier ones suggests that management effectiveness and programme implementation quality may be more influential than infrastructure alone. Future research incorporating health facility assessment data and district-level programme indicators could quantify these system-level determinants more precisely.” (See Discussion, Regional Disparities)

**Comment 6: Evaluation of healthcare infrastructure in low-performing regions**

*Future work should evaluate healthcare infrastructure in underperforming provinces.*

**Response:** We have substantially expanded the Future Research section to incorporate this suggestion alongside other research priorities. The revised section now explicitly recommends: (a) longitudinal cohort studies tracking individual children through the vaccination schedule; (b) implementation research comparing high-performing and low-performing regions to identify scalable best practices; (c) evaluation of healthcare infrastructure and resource allocation in regions falling significantly below national coverage averages; (d) qualitative investigation of zero-dose children concentrated in disadvantaged populations; and (e) purpose-built studies of MR2 performance using age-appropriate denominators. (See Future Research Directions)

**JOURNAL REQUIREMENTS**

**Requirement 1: Data Availability Statement**

*You indicated that your data is available only on request from a third party. Please provide the name of the third party contact or contact details.*

**Response:** We have updated the Data Availability Statement in the Editorial Manager online form to include the third-party contact details: “The data underlying this study are publicly available from the Demographic and Health Surveys (DHS) Program. Researchers can access the Ghana 2022 DHS dataset by registering at https://dhsprogram.com and submitting a data access request. Contact: The DHS Program, ICF International, 530 Gaither Road, Suite 500, Rockville, MD 20850, USA (info@dhsprogram.com).”

**Requirement 2: PLOS ONE style compliance**

*Please ensure the manuscript meets PLOS ONE style requirements, including title case, reference formatting, and other journal-specific guidelines.*

**Response:** We have reviewed the manuscript against PLOS ONE formatting requirements and made the following adjustments: (a) title and short title converted to sentence case per PLOS ONE style; (b) reference list verified for completeness and formatted per Vancouver style; (c) continuous line numbers added; (d) tables embedded within manuscript body at first mention; (e) heading styles applied consistently throughout.

**Requirement 3: Reference list review**

*Review reference list for completeness, retracted papers, and evaluate reviewer-cited references.*

**Response:** We identified that references 6 and 17 were identical (Gebeyehu NA et al., 2022). Reference 17 has been replaced with a distinct and contextually appropriate citation: Wariri O, Edem B, Nkereuwem E, Nkereuwem OO, Umeh G, Clark E, et al. Tracking coverage, dropout and multidimensional equity gaps in immunisation systems in West Africa, 2000–2017. BMJ Glob Health. 2019;4(5):e001713. All in-text citations have been updated accordingly. No retracted papers were identified in the reference list.

**ADDITIONAL CORRECTIONS**

In addition to addressing the above comments, we made the following corrections identified during the revision process:

**1. Numerical updates:** All estimates throughout the manuscript (Abstract, Results, Discussion, Conclusions) have been updated to reflect the corrected analysis. Key changes include: full immunization 63.9% → 69.5%; partial immunization 29.3%/31.2% → 25.7% (resolving a prior abstract–table inconsistency); MR1→MR2 dropout 37.5% → 28.9% (age-corrected); concentration index 0.061 → 0.045; regional gap 32.0pp → 35.7pp; education gap 17.2pp → 18.3pp.

**2. Regression coefficients updated:** Adjusted odds ratios were recalculated following the revised full immunization definition: wealth rich tertile aOR 1.43 → 1.39 (95% CI: 1.04–1.87); maternal education aOR 1.35 → 1.34 (95% CI: 1.04–1.72); ANC 4+ visits aOR 1.64 → 1.55 (95% CI: 1.19–2.01); facility delivery aOR 1.81 → 1.43 (95% CI: 1.08–1.90). Media exposure and distance to facility were no longer statistically significant in the fully adjusted model.

**3. R version updated:** The Methods section now correctly states R version 4.5.1 (previously listed as 4.3.1).

We believe these revisions address all concerns raised by the Editor and Reviewer. The manuscript has been substantially strengthened by the correction of the MR2 age-eligibility issue, the addition of clearer methodological descriptions, and the expanded discussion of regional determinants and future research priorities. We are grateful for the opportunity to revise this manuscript and hope it is now suitable for publication in PLOS ONE.

Sincerely,

Ahlam Tunteeya Saani, BDS, MPH

Goldfield Edem Azumah, MPH

*Corresponding author: Ahlam Tunteeya Saani, University of Ghana Dental School, College of Health Sciences, University of Ghana. Email: ahlamsaani@icloud.com*
